# Diagnostic utility of whole exome sequencing in children with suspected genodermatoses: a single-center retrospective study

**DOI:** 10.3389/fmed.2026.1932248

**Published:** 2026-10-07

**Authors:** Aliya A. Kudakaeva, Olga P. Parshina, Alexandra S. Botkina, Elvira R. Gumennaia, Vera A. Belova, Iuliia A. Vasiliadis, Natalia G. Ponikarovskaya, Alina F. Samitova, Anna O. Shmitko, Dmitriy O. Korostin

**Affiliations:** 1 Genomics Laboratory, Institute of Translational Medicine, Pirogov Russian National Research Medical University, Moscow, Russia; 2 Department of Dermatology, Russian Children’s Clinical Hospital, Moscow, Russia

**Keywords:** genetic diagnosis, genodermatoses, hereditary diseases, inherited skin disorders, WES, whole exome sequencing

## Abstract

**Introduction:**

Genodermatoses are a group of monogenic inherited skin disorders characterized by marked genetic heterogeneity. Although the phenotype of many genodermatoses is readily recognizable on clinical examination, it is often insufficient to establish the precise molecular genetic etiology. Currently, the use of whole exome sequencing in pediatric dermatology is considered preferable because of its accessibility and higher diagnostic accuracy compared with targeted sequencing approaches.

**Methods:**

Singleton whole exome sequencing was performed in 18 children (10 boys and 8 girls) aged 1–15 years with suspected genodermatoses. Before whole exome sequencing, all patients underwent dermatological evaluation, including a comprehensive phenotypic assessment, to determine indications for whole exome sequencing.

**Results:**

Previously described and novel pathogenic or likely pathogenic variants and copy number variation in 10 genes associated with genodermatoses were identified in 9 of the 18 patients (50%). In an additional 2 of the 18 patients, genetic variants were identified in 2 genes associated with other inherited disorders. These were Noonan syndrome, type 4, and pseudohypoaldosteronism, type IB2. The referral diagnosis was concordant with the whole exome sequencing findings in 6 of the 11 cases with pathogenic or likely pathogenic variants (54.54%). Variants of uncertain significance were identified in 4 patients. Two previously unreported likely pathogenic variants were detected in the JUP and SCNN1B genes. Previously reported variants were identified in *SOS1, FLG, JUP, KRT10, SPINK5, LEMD3*, and *NIPAL4*. Overall, 17 sequence variants were detected, including 6 pathogenic, 7 likely pathogenic, and 4 of uncertain clinical significance. In addition, one pathogenic deletion involving the STS gene was identified. In one patient with Netherton syndrome, the molecular diagnosis enabled the initiation of targeted pathogenic therapy. No disease–causing genetic variants were identified in 3 patients.

**Conclusion:**

Comprehensive phenotypic assessment combined with whole exome sequencing represents a useful diagnostic tool in pediatric dermatology, enabling rapid diagnosis in clinically challenging and undifferentiated cases. Furthermore, this approach facilitates the identification of pathogenic variants not only in genes associated with inherited skin disorders but also in other clinically relevant genes consistent with the suspected diagnosis.

## 1 Introduction

Genodermatoses constitute a genetically heterogeneous group of monogenic inherited skin disorders (1). These conditions are relatively common among hereditary diseases, with an estimated prevalence ranging from 1:250 to 1:1,000,000 (2). Consequently, dermatologists and clinical geneticists are likely to encounter patients with these disorders during their clinical practice on multiple occasions (3).

Although the clinical phenotype of many genodermatoses is readily recognizable, substantial phenotypic overlap often complicates differential diagnosis, making it difficult to establish the precise molecular genetic cause during the initial clinical evaluation (1).

Most genodermatoses manifest during the first years of life. Early diagnosis is essential for implementing a personalized approach to patient management. Certain inherited skin disorders, such as xeroderma pigmentosum, are associated with an unfavorable prognosis in the absence of timely treatment and appropriate long-term surveillance and may be complicated by the development of malignant neoplasms (3). Even relatively common disorders, including the ichthyoses, markedly impair patients’ quality of life. Epidermal barrier dysfunction predisposes affected individuals to recurrent infections and hypohidrosis and may also be accompanied by ocular abnormalities, hearing impairment, and nutritional disorders (4). For some inherited skin diseases, such as epidermolysis bullosa, gene therapy has already been developed (5), emphasizing the importance of establishing an accurate diagnosis at an early stage to enable appropriate therapeutic intervention.

Currently, the use of whole exome sequencing (WES) in pediatric dermatology is considered preferable because of its accessibility, higher diagnostic accuracy compared with targeted sequencing approaches, and ability to identify novel disease-associated genes (6). It is a useful diagnostic tool for genetically heterogeneous disorders and for identifying multiple inherited disorders in a single patient (6).

In the present study, we report the clinical and genetic findings obtained in 18 unrelated probands presenting with a spectrum of inherited skin disorders and other inherited disorders.

## 2 Patients, materials and methods

A total of 18 patients aged 1–15 years were enrolled in this retrospective study. The mean age was 8.88 ± 3.76 years; the cohort comprised 10 boys and 8 girls. All patients were consecutively admitted to the Department of Dermatology of the Russian Children's Clinical Hospital between June 2023 and December 2025 and were evaluated by a dermatologist before WES. The dermatologist performed a comprehensive clinical assessment and determined the indications for WES, which included clinical features suggestive of an inherited disorder such as severe or chronic disease, resistance to standard therapy, early disease onset, associated comorbidities, or a characteristic phenotype raising suspicion of a specific genetic condition.

The study consisted of a retrospective analysis of data obtained during routine clinical practice. No additional diagnostic procedures, repeat WES, collection of biological samples, or changes in patient management were performed beyond standard medical care and routine diagnostic procedures prior to the initiation of this analysis. No prospective patient recruitment was undertaken. Inclusion criteria were: (a) pediatric patients followed in the Department of Dermatology of the Russian Children's Clinical Hospital between June 2023 and April 2024; (b) clinical indication for genetic testing due to a suspected inherited skin disorder; and (c) availability of a blood sample, basic clinical data, and signed informed consent. There were no additional exclusion criteria beyond insufficient sample quality, incomplete clinical information, or lack of informed consent.

The study was conducted in accordance with the Declaration of Helsinki. This study was approved for publication by the Ethics Committee of Pirogov Russian National Research Medical University.

All WES analyses were completed by April 2024. Reclassification of variants and review of previously inconclusive reports were performed in March 2026 as part of routine laboratory practice.

Exome sequencing of genomic DNA extracted from the patient's peripheral blood sample was carried out using the Agilent SureSelect Human All Exon V8 baits at the Genomics Laboratory of Pirogov Russian National Research Medical University, following the RSMU_exome protocol (7). The DNA library was prepared using the MGIEasy Universal DNA Library Prep Set, enriched and sequenced in the PE100 mode on the DNBSEQ-G400 platform (7).

The quality of fastq files was analysed using FastQC v0.12.1 (8). Based on the quality metrics, the fastq files were trimmed using BBDuk by BBMap v38.96 (9). Reads were aligned to the indexed reference genome GRCh38.p14 using bwa-mem2 v2.2.1 (10). SAM files were converted into BAM files and sorted using SAMtools v1.9 (11). The number of duplicates was marked using Picard MarkDuplicates v2.22.4. (12). Variant calling was performed using bcftools v1.9 (13) and DeepVariant v1.5.0 (14). After variant calling, multi-allelic variants in VCF files were decomposed into bi-allelic variants using vt decompose v0.5772 (15) and then normalized using vt normalize v0.5772. Then, the VCF files were filtered by the target enrichment regions defined in a BED file. A depth coverage filter (DP ≥ 3) was applied to variants called by both programs. An additional filter (FILTER=PASS) was applied to variants called by DeepVariant, after which VCF files were merged using bcftools-1.9 merge. The resulting variants were annotated using ANNOVAR (16). CNV analysis was performed using CNVkit v0.9.8 (17). The resulting VCF files containing the list of CNVs were converted into BED format and annotated using ClassifyCNV v1.1.1 (18) and AnnotSV v3.3.8 (19), and the Franklin variant interpretation platform.

Clinical interpretation of genetic variants was performed according to the recommendations of the American College of Medical Genetics and Genomics (ACMG) (20) (21). The sequencing depth for all analyzed variants was at least 14 ×. The allele frequency threshold was set at ≤0.01 in the gnomAD v4.1.1 (22) and RUSeq (23) population databases (genome and exome datasets) at the time of variant interpretation.

Variant prioritization was carried out using the laboratory's pathogenicity-filtering pipeline. This included identification of variants classified in ClinVar as pathogenic or likely pathogenic, as well as variants predicted to result in loss of protein function, including nonsense variants, frameshift variants, and canonical splice-site alterations. Subsequently, variants were filtered using the Human Phenotype Ontology (HPO) term “Abnormal skin morphology” (HP:0011121) (24). Only variants with a REVEL score >0.7 (25) and a SpliceAI score >0.2 (26) were retained for further analysis.

## 3 Results

The referral diagnoses included ichthyosis (5 patients, 27.8%), Olmsted syndrome (2 patients, 11.1%), epidermolytic hyperkeratosis (2 patients, 11.1%), Netherton syndrome (2 patients, 11.1%), pityriasis rubra pilaris, pityriasis lichenoides chronica, ectodermal dysplasia, psoriasis, Buschke-Ollendorff syndrome, palmoplantar keratoderma, and acanthosis nigricans with a verrucous nevus (one case each). Concomitant conditions included pseudohypoaldosteronism, arthritis with joint contractures, scoliosis, umbilical hernia, sensorineural hearing loss, myopia, hyperopia, nystagmus, autoimmune thyroiditis, and cryptorchidism (one case each). Epilepsy was identified in two patients, and two patients had atopic dermatitis. Descriptions of the variants, their classifications, and the criteria applied are provided in Table 1 and Supplementary Material 2.

**Table 1:** Clinical characteristics of the patients and genetic variants identified by WES.

Patient	Age, years	Sex	Clinical diagnosis	Coincidence of clinical and genetic diagnosis	Genetic diagnosis, OMIM	Variant (hg 38)	Classification of variant/CNV	Zygosity	Reported	Allele frequency in gnomAD v4.1.1 (exomes + genomes)	Allele frequency in RUSeq
Patient 1	8	M	Ectodermal dysplasia	No	Noonan syndrome, 4 (610733)	*SOS1*(NM_005633.4):c.1654A > G (p.Arg552Gly)	P	het	Yes (37)	0.000001859	-
Patient 2	10	M	Pityriasis rubra pilaris	No	Epidermolysis bullosa simplex 5A, Ogna type (131950)	*PLEC*(NM_201378.4):c.12544G > A (p.Asp4182Asn)	VUS	het	No	0.00001550	0.0001043
Patient 3	9	F	Olmsted syndrome	No	Ichthyosis vulgaris (146700)	*FLG*(NM_002016.2):c.7339C > T (p.Arg2447Ter)	LP	het	Yes (38)	0.003890	0.004266
					Naxos disease (601214)	*JUP*(NM_002230.4):c.468G > A (p.Pro156=)	P	het	Yes (39), (27)	0.000006209	0.0001050
						*JUP*(NM_002230.4):c.187C > T (p.Gln63Ter)	LP	het	No	-	-
Patient 4	15	M	Olmsted syndrome	Yes	X-linked Olmsted syndrome (300918)	*MBTPS2*(NM_015884.4):c.656G > C (p.Arg219Thr)	VUS	hemi	No	-	-
Patient 5	15	F	Epidermolytic hyperkeratosis	Yes	Autosomal dominant epidermolytic hyperkeratosis 2A (620150)	*KRT10*(NM_000421.5):c.467G > A (p.Arg156His)	P	het	Yes (40)	-	0.0001040
Patient 7	9	M	Epidermolytic hyperkeratosis	Yes	Epidermolytic hyperkeratosis, 1 (113800)	*KRT1*(NM_006121.4):c.583A > T (p.Ile195Phe)	VUS	het	No	-	-
Patient 8	14	M	Netherton syndrome	Yes	Netherton syndrome (256500)	*SPINK5*(NM_006846.4):c.1048C > T (p.Arg350Ter)	P	hom	Yes (28)	0.00004776	0.0001073
Patient 9	7	M	Ichthyosis	Yes	X-linked ichthyosis (308100)	Deletion with genomic coordinates chrX:7050166-7804358	P	hemi	Yes (32)	-	-
Patient 10	11	F	Ichthyosis	Yes	Autosomal recessive congenital ichthyosis, 6 (612281)	*NIPAL4*(NM_001099287.2):c.586G > A (p.Gly196Arg)	VUS	hom	No	-	-
Patient 11	1	F	Pseudohypoaldosteronism, type I. Congenital ichthyosis?	No	Pseudohypoaldosteronism, type IB2, autosomal recessive (620125)	*SCNN1B*(NM_000336.3):c.978C > A (p.Tyr326Ter)	LP	hom	No	0.000001859	0.0001041
Patient 12	8	F	Ichthyosis	Yes	Ichthyosis vulgaris (146700)	*FLG*(NM_002016.2):c.4420C > T (p.Arg1474Ter)	LP	het	Yes (38)	0.0003533	0.0002082
						*FLG*(NM_002016.2):c.10558C > T (p.Gln3520Ter)	LP	het	Yes (41)	0.00002048	-
Patient 13	8	M	Ichthyosis	No	Netherton syndrome (256500)	*SPINK5*(NM_006846.4):c.1048C > T (p.Arg350Ter)	P	hom	Yes (28)	0.00004776	0.0001073
Patient 15	9	M	Buschke-Ollendorff syndrome	Yes	Buschke-Ollendorff syndrome (166700)	*LEMD3*(NM_014319.5):c.1963C > T (p.Arg655Ter)	P	het	Yes (42)	0.000002482	-
Patient 16	8	F	Ichthyosis	Yes	Autosomal recessive congenital ichthyosis, 6 (612281)	*NIPAL4*(NM_001099287.2):c.341C > A (p.Ala114Asp)	LP	hom	Yes (43)	0.001112	0.0003149
Patient 18	1	F	Psoriasis	No	Ichthyosis vulgaris (146700)	*FLG*(NM_002016.2):c.3059C > G (p.Ser1020Ter)	LP	het	Yes (44)	0.0000855	0.0009365

F, female; M, male; P, pathogenic; LP, likely pathogenic; VUS, variant of uncertain significance; het, heterozygous; hom, homozygous; hemi, hemizygous.

### 3.1 Genetic variants detected in patients

Previously described and novel pathogenic or likely pathogenic variants and copy number variation in 10 genes associated with genodermatoses were identified in 9 of the 18 patients (50%). In an additional 2 of the 18 patients, pathogenic or likely pathogenic variants were identified in 2 genes associated with other inherited disorders that include skin involvement as part of their clinical manifestations. These were Noonan syndrome, type 4, and pseudohypoaldosteronism, type IB2. The referral diagnosis was concordant with the WES findings in 6 of the 11 cases with pathogenic or likely pathogenic variants (54.54%) (Figure 1). Genetic variants were identified in 15 of the 18 patients (83.33%). Variants of uncertain significance (VUS) were identified in 4 of these patients. No isolated carrier status for pathogenic variants associated with genodermatoses was identified.

**Figure 1:**
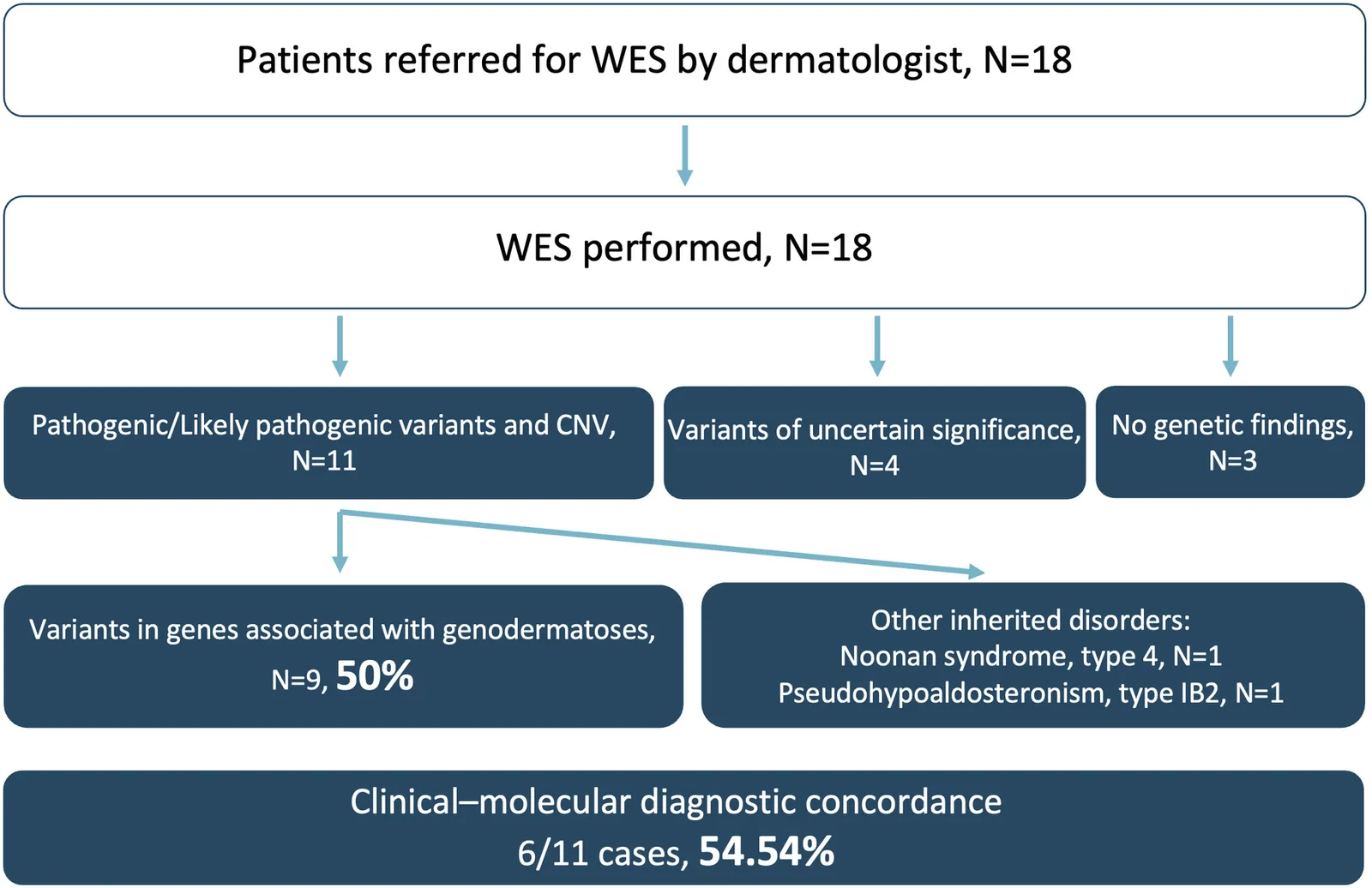
Study design and diagnostic outcomes. N – number of patients; WES – whole exome sequencing; CNV – copy number variation.

The most frequently identified variants were located in the *FLG* gene. Four pathogenic or likely pathogenic variants were detected in 3 patients (patients 3, 12, and 18) (Table 1).

In patient 3, a variant *FLG*(NM_002016.2):c.7339C > T (p.Arg2447Ter) co-occurred with two heterozygous variants in the *JUP* gene (*JUP*(NM_002230.4):c.468G > A (p.Pro156=) and *JUP*(NM_002230.4):c.187C > T (p.Gln63Ter)). She was initially diagnosed with Olmsted syndrome; however, WES established the coexistence of two distinct inherited disorders: ichthyosis vulgaris (OMIM: 146700) and Naxos disease (OMIM: 601214). A detailed description of the status localis and representative clinical photographs of the cutaneous lesions are provided in Supplementary Material 1. In addition to cutaneous manifestations, Naxos disease is associated with an increased risk of arrhythmogenic right ventricular cardiomyopathy and life-threatening cardiac arrhythmias. The *JUP* (NM_002230.4):c.468G > A (p.Pro156=) variant has been reported to reduce plakoglobin levels in the skin of affected individuals, possibly by disrupting splicing (27). The SpliceAI score for this variant is 0.87 (26).

Two unrelated patients (patients 8 and 13) harbored the previously reported homozygous pathogenic variant *SPINK5*(NM_006846.4):c.1048C > T (p.Arg350Ter) in the *SPINK5* gene, which is associated with Netherton syndrome (OMIM: 256500) (28). Both patients exhibited a highly similar clinical phenotype characterized by fine scaling on a background of diffuse erythroderma. In addition, patient 13 presented with bilateral cryptorchidism. This patient received pathogenetic treatment with secukinumab at a dose of 75 mg administered subcutaneously. A positive therapeutic effect was observed, manifested by a reduction in erythema and scaling. It is known that some genodermatoses, including Netherton syndrome and congenital ichthyosiform erythroderma, are characterized by a marked increase in circulating interleukin-17 levels and IL-22-producing T cells (29). Secukinumab, a monoclonal antibody targeting IL-17, has been shown to produce substantial clinical improvement in patients with Netherton syndrome (30).

Patient 11 was referred with suspected ichthyosis and atopic dermatitis. She had a significant family history, with a sibling who had died of hypoaldosteronism. Shortly after birth, the proband presented with moderate erythroderma, marked xerosis, and generalized skin scaling, all of which recurred persistently during follow-up. Beginning on the fourth day of life, severe hyperkalemia (up to 8.3 mmol/L) and hyponatremia (down to 114 mmol/L) developed, complicated by cardiac arrhythmia, bradypnea, and hyperlactatemia. Neither infusion therapy nor hormonal treatment resulted in clinical improvement. Based on the clinical findings, pseudohypoaldosteronism was diagnosed. Seborrheic dermatitis was diagnosed at 3 months of age, followed by atopic dermatitis at 12 months, at which time ichthyosis was also suspected. WES identified a previously unreported homozygous *SCNN1B*(NM_000336.3):c.978C > A (p.Tyr326Ter) variant. Biallelic variants in *SCNN1B* are associated with autosomal recessive pseudohypoaldosteronism type IB2 (OMIM: 620125). *SCNN1B* gene has not been associated with genodermatoses but may provide a molecular explanation for the patient's cutaneous phenotype. In this condition, cutaneous rash, pustular eruptions, and miliaria rubra–like lesions are frequently observed (31). No genetic variants associated with ichthyosis or atopic dermatitis were detected.

A copy number variation (CNV), consisting of a hemizygous deletion of approximately 754 kb at the Xp22.31 region, was identified in patient 9, a male with ichthyosis, following manual inspection using the Integrative Genomics Viewer (IGV). The genomic coordinates of this deletion are chrX:7050166-7804358. Complete absence of read coverage was observed across the deleted region, supporting the presence of a hemizygous deletion. The deletion was not confirmed by an independent method, such as MLPA, chromosomal microarray, or qPCR. The deleted region encompassed the *STS, PUDP,* and *MIR4767* genes. Deletions of the *STS* gene are a well-established cause of X-linked ichthyosis (OMIM: 308100) (32).

No variants associated with inherited skin disorders were identified in 3 patients. This subgroup included patients with pityriasis lichenoides chronica, palmoplantar keratoderma, and acanthosis nigricans with papillomatosis.

Overall, WES identified two previously unreported likely pathogenic variants in the *JUP* and *SCNN1B* genes, together with previously reported variants in the *SOS1, FLG, JUP, KRT10, SPINK5, LEMD3*, and *NIPAL4* genes. In total, 17 sequence variants were detected, including 6 pathogenic variants, 7 likely pathogenic variants, and 4 VUS (Figure 2). By variant type, the following were identified: 7 missense variants, 9 nonsense variants, and 1 synonymous variant (Figure 3). In addition, one pathogenic deletion involving the *STS* gene was identified.

**Figure 2:**
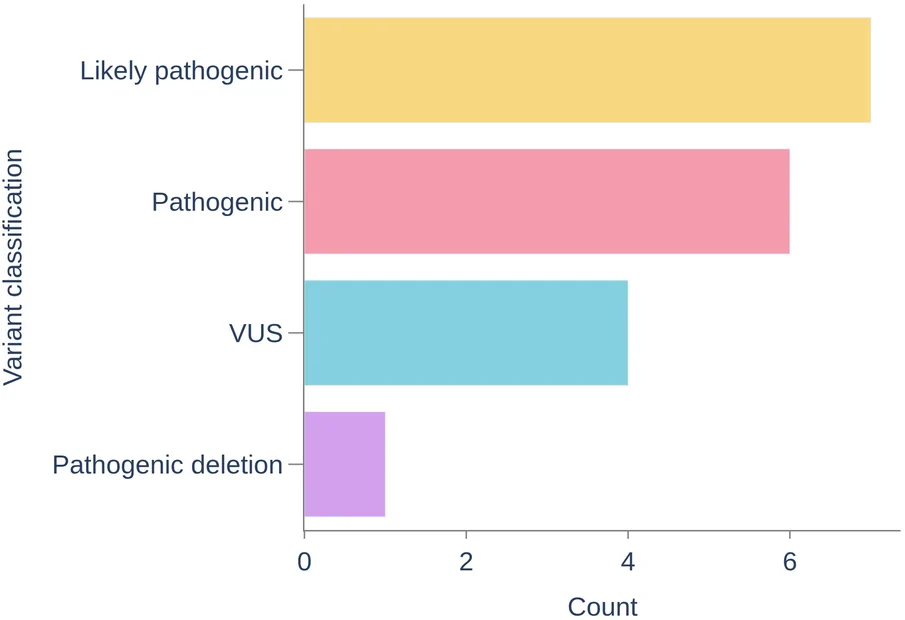
Distribution of genetic variants and CNVs according to the ACMG/AMP classification: 6 pathogenic variants, 7 likely pathogenic variants, 4 variants of uncertain significance (VUS), 1 pathogenic copy number variation (CNV) - deletion.

**Figure 3:**
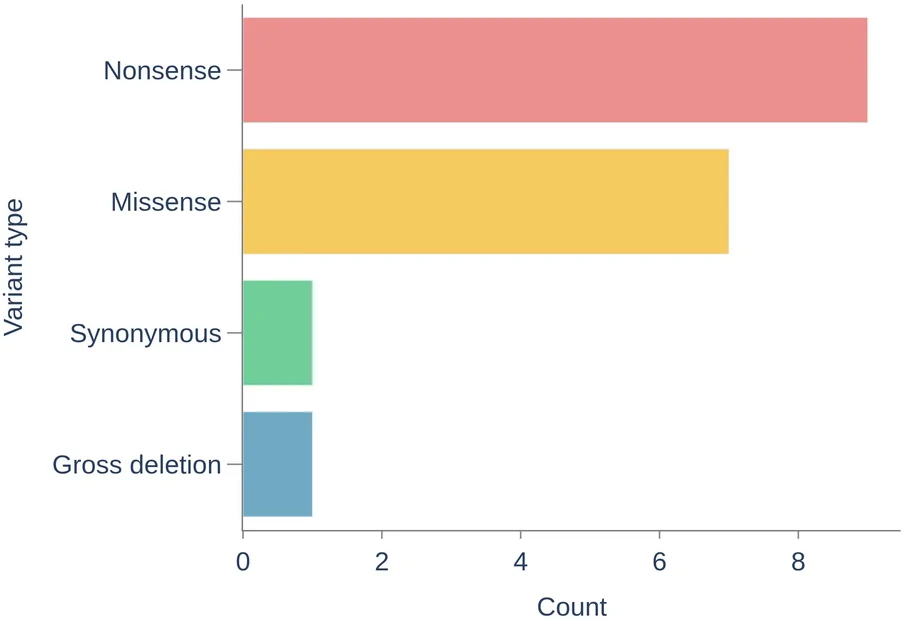
Distribution of genetic variants and CNV by molecular type: 9 nonsense variants, 7 missense variants, 1 synonymous variant, 1 genomic deletion.

A total of 12 genetic disorders were detected in 11 patients. Among these, 5 disorders were inherited in an autosomal dominant manner, 1 was X-linked, and 6 were autosomal recessive. Among the autosomal recessive cases, 4 patients had homozygous variants, whereas 2 patients had putative compound heterozygous variants. One patient was diagnosed with two coexisting genetic disorders, one autosomal recessive and one autosomal dominant.

A detailed description of the status localis for all patients, together with the results of skin biopsy and representative clinical photographs of the cutaneous lesions, is provided in Supplementary Material 1. Comprehensive information on the genetic variants identified by WES is presented in Table 1 and Supplementary Material 2.

## 4 Discussion

WES has proven to be a useful diagnostic tool in pediatric dermatology. The application of WES enabled the identification of pathogenic or likely pathogenic variants in genes associated with genodermatoses in 9 of the 18 patients (50%). Our findings are comparable to those reported in similar studies worldwide (1). However, the study cohorts differ in the representation of specific groups of genodermatoses. In our cohort, there were no patients with tumor predisposition syndromes or albinism.

In 5 patients, the genetic findings identified by WES did not correspond to the initial referral diagnosis, resulting in a revision of the diagnosis in 45.45% of cases. The discordance between the referral diagnosis and genetic findings may be explained by the phenotypic overlap among inherited skin disorders and other inherited disorders, further underscoring the diagnostic value of WES.

The most common disease in our cohort was ichthyosis, identified in 5 patients. Among these, 3 patients were diagnosed with *FLG*-associated ichthyosis vulgaris, one patient with autosomal recessive congenital ichthyosis, type 6, and one patient with X-linked ichthyosis. All variants identified in the *FLG* gene were nonsense variants. Importantly, the accurate clinical diagnosis of ichthyosis in patient 9 prompted targeted evaluation of the *STS* gene. This finding highlights the importance of CNV analysis in patients with a referral diagnosis of ichthyosis. In some cases, WES data require additional manual inspection using the IGV. Given that *STS* deletions represent the most common cause of X-linked ichthyosis (32), the *STS* gene should be specifically evaluated in IGV when no pathogenic single-nucleotide variants are identified.

WES can identify variants in other clinically relevant genes that were not included in the differential diagnostic workup at the clinical stage. In our study, these included the *SOS1* and *SCNN1B* genes. Performing WES in patient 11 allowed us to identify a potentially life-threatening condition – pseudohypoaldosteronism. Notably, the patient's initial manifestations were exclusively cutaneous; however, no pathogenic variants in genes associated with genodermatoses were identified.

WES not only allowed us to identify the cause of the disease but also to plan further clinical follow-up, determine the risks of extracutaneous complications, as illustrated by the patients with Noonan syndrome, Naxos disease, and pseudohypoaldosteronism in the present study, and initiate pathogenetic treatment when available, as illustrated by the patient with Netherton syndrome. The effectiveness of genetic counseling for the families was also increased. Thus, in 5 patients with autosomal dominant disorders, the risk to future offspring may reach 50%. In the family of patient 11, pseudohypoaldosteronism occurred for the second time. A search for the genetic cause of the disease was not performed in the affected sibling. However, a variant was identified in our proband, which will allow to plan future pregnancies, taking the associated risks into account after undergoing genetic testing. In cases of X-linked and autosomal recessive inheritance, parental testing is required to determine the recurrence risks.

An important advantage of WES is the relatively short time required to obtain a diagnosis (6). In our study, the mean turnaround time for WES analysis was 29.33 calendar days.

During the study, 4 VUS were identified in the *PLEC*, *MBTPS2, KRT1*, and *NIPAL4* genes. In 3 patients, the initial diagnosis was concordant with the genetic findings. Nevertheless, insufficient evidence regarding the pathogenicity of these variants did not allow a definitive diagnosis to be established in our patients. Additional studies are required to confirm or refute the significance of these variants, including segregation analysis and functional studies.

A genetic variant could not be identified in 3 patients, including children with pityriasis lichenoides chronica, palmoplantar keratoderma, and acanthosis nigricans with papillomatosis.

Previous studies have reported that the diagnostic yield of WES or whole genome sequencing in patients with palmoplantar keratoderma may reach 83%, although the diagnostic rate is lower in patients with diffuse (33) and focal (34) forms of the disease. Patient 14 in the cohort presented with palmoplantar keratoderma. Following a negative WES result, a skin biopsy was performed, which revealed cutaneous calcinosis. Surgical treatment was subsequently undertaken: the skin lesions were initially excised surgically, and five months later, the remaining lesions were treated with laser therapy. During the interval between hospital admissions, small indurations were noted at the sites of the lesions previously treated with laser therapy. The remaining lesions spontaneously flattened without any treatment.

Acanthosis nigricans may occur as a manifestation of acquired disorders, such as obesity-associated insulin resistance, or as a part of hereditary syndromic conditions. It has been described in association with lipodystrophies, *FGFR*-associated disorders, and several other genetic syndromes, although isolated hereditary forms have also been reported (35). To date, the diagnostic performance of molecular genetic testing in patients with acanthosis nigricans has not been systematically evaluated. Furthermore, according to the OMIM database, isolated acanthosis nigricans has not yet been associated with any causative gene (OMIM %100600). Following a negative WES result, patient 17 underwent surgical treatment consisting of photovaporization and photothermolysis of the pigmentation in the collar area. Five months later, the papillomatous lesions in the right axillary region were surgically excised. Histological examination of the surgical specimen confirmed the diagnosis of acanthosis nigricans form of epidermal nevus. No sequencing of the nevus tissue was performed.

Pityriasis lichenoides chronica belongs to the spectrum of autoinflammatory skin disorders, which may have either multifactorial or monogenic etiologies. Monogenic forms have been associated with pathogenic variants in *IL36RN, CARD14, AP1S3, IL1RN, MVK, MVD, PMVK, FDPS, JAK1, POMP, NLRP1*, and *EGFR* genes (36)*.* However, the precise etiology of pityriasis lichenoides chronica remains unknown. Following a negative WES result, a skin biopsy was performed in patient 6, which led to the diagnosis of pityriasis lichenoides chronica. Methotrexate therapy was initiated. Data on the effects of the treatments administered are unavailable.

Our study was retrospective and single-center, and the small number of patients limited the sample size. This represents a limitation that may have influenced the results obtained. Larger multicenter studies are required to confirm our findings.

Additional limitations include the absence of segregation analysis. This precluded determination of the phase (cis or trans) of the variants and confirmation of compound heterozygosity, as well as definitive confirmation of homozygosity and determination of the presence or absence of a deletion on the second allele. The ability to detect a deletion involving the second allele is a limitation of singleton WES. For previously undescribed VUS, segregation analysis is required to confirm or refute their clinical significance. Establishing a *de novo* status may provide additional evidence supporting the pathogenicity of the identified variants. Furthermore, WES is limited to protein-coding sequences and adjacent intronic regions. Therefore, it does not allow the investigation of deep intronic, regulatory, and intergenic regions or gene expression, tandem repeat expansions, or mitochondrial DNA. WES also presents challenges in the detection of structural variants, including translocations, inversions, deletions, and duplications. Since the analyzed biomaterial was venous blood, challenges in detecting somatic genetic variants should also be considered among the limitations.

## 5 Conclusion

Our findings demonstrate that whole exome sequencing, when combined with comprehensive phenotypic assessment, represents a useful diagnostic tool in pediatric dermatology. This approach facilitates rapid diagnosis in clinically challenging and undifferentiated cases while enabling the identification of pathogenic variants not only in genes associated with inherited skin disorders but also in genes relevant to the patient's suspected underlying condition. Furthermore, establishing a precise diagnosis may directly influence clinical management by enabling pathogenetic therapy, as illustrated by the patient with Netherton syndrome in the present study.

## Data Availability

The raw data supporting the conclusions of this article will be made available by the authors upon reasonable request.
