# Supplementary material for: Diagnostic utility of whole exome sequencing in children with suspected genodermatoses: a single-center retrospective study
Source: Front Med (Lausanne). 2026 Oct 7;13:1932248. doi: 10.3389/fmed.2026.1932248 (PMC13682972; doi:10.3389/fmed.2026.1932248)
Supplement: Supplementary file 1 [file Supplementaryfile1.docx]

***Supplementary materials 1***

Clinical photographs, clinical diagnoses, genetic variants, and physical examination findings are presented below, including a concise description of the status localis extracted from the patient’s medical records.

Written informed consent for the publication of anonymized clinical data and the use of clinical photographs for scientific research, scientific publications, and educational purposes (including the training of medical students and residents) was obtained from the legal guardians of all patients. Clinical photographs are available for a subset of patients.

**Patient 1**

*SOS1*(NM_005633.4):c.1654A>G (p.Arg552Gly), heterozygous.

Clinical diagnosis: Hidrotic ectodermal dysplasia. Generalized epilepsy. Strabismus.

Status localis: The scalp was devoid of terminal hair, with a single focal area of vellus hair growth in the occipital region. The scalp skin was of normal physiological color. Hair follicles were largely inconspicuous, with focal preservation. Eyebrows and eyelashes were sparse, and vellus hairs were absent. Fingernails and toenails demonstrated transverse ridging.

Clinical photographs: Not available.

**Patient 2**

*PLEC*(NM_201380.4):c.12997G>A p.Asp4333Asn, heterozygous.

Clinical diagnosis: Pityriasis rubra pilaris. Atopic dermatitis.

Status localis: Multiple grouped follicular papules of erythematous to yellowish coloration were distributed symmetrically over the face, trunk, extensor surfaces of the extremities, palms, and soles. The papules were covered with adherent hyperkeratotic scales, producing the characteristic “nutmeg grater” sign. In addition, moderately lichenified plaques were present within the antecubital and popliteal fossae.

Skin biopsy: Histopathological examination demonstrated mild hyperkeratosis, hypergranulosis, and acanthosis of the epidermis with focal erosions. The superficial dermis showed prominent subepidermal lymphohistiocytic inflammatory infiltrates containing a small number of eosinophils. Immunohistochemical analysis revealed that the inflammatory infiltrates consisted predominantly of CD3-positive T lymphocytes, with scattered CD20-positive B lymphocytes and CD117-positive mast cells. Numerous antigen-presenting cells were identified, including intraepidermal dendritic Langerhans cells and activated histiocytes within the inflammatory infiltrate, exhibiting immunoreactivity for S100, Langerin (CD207), and CD1a. Histopathological diagnosis: Pityriasis rubra pilaris.

**Patient 3**

*FLG*(NM_002016.2):c.7339C>T (p.Arg2447Ter), heterozygous,

*JUP*(NM_002230.4):c.468G>A (p.Pro156=), heterozygous,

*JUP*(NM_002230.4):c.187C>T (p.Gln63Ter), heterozygous.

Clinical diagnosis: Olmsted syndrome.

Status localis: Terminal hair was completely absent from the scalp, face, trunk, and extremities. The glabrous skin exhibited marked xerosis with numerous follicular papules, particularly involving the abdomen and extremities. The “nutmeg grater” sign was strongly positive. Pronounced hyperkeratosis on a background of moderate erythema and cutaneous infiltration was observed in the major flexural folds, over the extensor surfaces of the extremities, and in the periorificial and perianal regions, accompanied by deep, painful fissures. Severe palmoplantar keratoderma was present, characterized by massive, well-demarcated hyperkeratotic plaques extending onto the fingers and toes. Dental abnormalities, nail dystrophy, flexion deformities of the digits, and generalized joint hypermobility were noted. Residual flaccid blisters were present on the buttocks. The patient reported pruritus and pain. The mucous membranes were unremarkable.

**Patient 4**

*MBTPS2*(NM_015884.4):c.656G>C (p.Arg219Thr), hemizygous.

Clinical diagnosis: Olmsted syndrome. Nystagmus. Congenital optic disc anomaly. Phimosis.

Status localis: Multiple papules, plaques, crusts, scales, and fissures were distributed over the scalp, face, trunk, palms, soles, and the skin surrounding all natural body orifices on a background of marked xerosis. The palms and soles demonstrated extensive yellow hyperkeratotic plaques with fissuring and scaling overlying erythematous, infiltrated skin. Fingernails and toenails were dystrophic. The fingers exhibited tapering deformities, with restricted range of motion in the distal interphalangeal joints. The anogenital region showed extensive crusting and fissuring associated with marked erythema and cutaneous infiltration. The flexural surfaces of the elbows and knees demonstrated pronounced lichenification together with sharply demarcated plaques measuring up to 10 cm in diameter, exhibiting coarse lamellar scaling, erythema, and infiltration. The oral mucosa and tongue contained dense white plaque-like lesions consistent with oral leukoplakia.

**Patient 5**

*KRT10*(NM_000421.5):c.467G>A (p.Arg156His), heterozygous.

Clinical diagnosis: Epidermolytic hyperkeratosis.

Status localis: The scalp was covered with fine lamellar scales and scale-crusts. The skin of the face, neck, trunk, and extremities was diffusely dry and covered with dense hyperkeratotic scales. Pronounced hyperkeratotic accumulations were present over the major joints, the dorsal surface of the hands, the ankles, and the feet. Areas of crust detachment were associated with superficial erosions, excoriations, and serohemorrhagic crusts. The nails were unaffected. Both vellus and terminal hair growth were preserved.

**Patient 6**

No genetic variants identified.

Clinical diagnosis: Pityriasis lichenoides chronica.

Status localis: Multiple discrete papules without a tendency to coalesce were observed on the hands, accompanied by isolated small, round erosions with well-defined regular borders, some of which were covered by crusts. On the anterior surface of the left lower leg, a round ulcer with sharply demarcated regular margins and a rim of moderate erythema was present. The perilesional skin contained scattered pink macules and papules without evidence of confluence or grouping. Areas of resolved lesions exhibited residual post-inflammatory hyperpigmentation. The uninvolved skin was unremarkable. Skin appendages were unaffected.

Skin biopsy: Histopathological examination revealed a skin specimen containing two foci of ulceration. The ulcer bases were composed of granulation tissue admixed with neutrophilic debris. The adjacent epidermis was mildly thickened and demonstrated irregular acanthosis. The dermis showed a moderate perivascular lymphocytic infiltrate composed predominantly of CD4-positive T lymphocytes, with fewer CD8-positive T lymphocytes and occasional CD20-positive B lymphocytes, accompanied by rare neutrophils and eosinophils. No histopathological evidence of vasculitis or neutrophilic dermatosis was identified. In conjunction with the clinical findings, the histopathological features were considered most consistent with pityriasis lichenoides et varioliformis acuta (PLEVA).

**Patient 7**

*KRT1*(NM_006121.4):c.583A>T (p.Ile195Phe), heterozygous.

Clinical diagnosis: Epidermolytic hyperkeratosis. Bilateral grade IV sensorineural hearing loss. Keratitis-ichthyosis-deafness (KID) syndrome? Epilepsy in complete clinical remission.

Status localis: Fine- to medium-sized lamellar scaling was present on the scalp and face. The palms and soles demonstrated extensive hyperkeratosis with deep fissures and erosions. Marked hyperkeratosis with coarse lamellar, crumb-like hyperkeratotic scales was observed within the major flexural areas, including the knees, elbows, wrists, ankles, neck, and axillae. The trunk and extremities exhibited multiple dirty gray hyperkeratotic plaques involving the glabrous skin. Hair was dry, sparse, and exhibited slow growth. Fingernails and toenails were thickened, yellowish, and showed pronounced onychogryphosis.

 

**Patient 8**

*SPINK5*(NM_006846.4):c.1048C>T (p.Arg350Ter), homozygous.

Clinical diagnosis: Netherton syndrome.

Status localis: Diffuse erythroderma involved the entire cutaneous surface. The palms and soles demonstrated marked hyperkeratosis with coarse lamellar scaling, whereas the face, trunk, and extremities exhibited fine lamellar desquamation. Bright erythema was present on the face, accompanied by large white lamellar scales in the perioral region. Multiple pustules were observed on the nasal skin. The conjunctivae were hyperemic, with associated ectropion. Dermoscopic examination revealed numerous bamboo hairs (trichorrhexis invaginata). Two nail plates (one fingernail and one toenail) were thickened, yellowish in color, and had irregular margins.

**Patient 9**

Del:chrX:7050166-7804358, hemizygous.

Clinical diagnosis: Ichthyosis vulgaris.

Status localis: Numerous densely adherent grayish-brown scales were distributed diffusely over the entire cutaneous surface.

Clinical photographs: Not available.

**Patient 10**

*NIPAL4*(NM_001099287.2):c.586G>A (p.Gly196Arg), homozygous.

Clinical diagnosis: Congenital ichthyosis. Juvenile idiopathic arthritis.

Status localis: The glabrous skin exhibited moderate-to-large lamellar scaling with focal areas of hyperkeratosis involving flexural regions and sites subjected to mechanical friction, accompanied by multiple excoriations. The palms and soles demonstrated mild hyperkeratosis and hyperlinearity with linear fissures. Numerous lentigines were present on the face and trunk, having developed during immunosuppressive therapy; dermoscopic examination revealed no atypical features. The scalp was covered by a small number of densely adherent yellowish crusts. Hair was dry but exhibited normal to accelerated growth. Distal onychogryphosis of the nail plates was present. Mild ectropion and deformity of the ears were also noted.

Musculoskeletal examination revealed flexion contractures of both the proximal and distal interphalangeal joints, most pronounced in the second, fourth, and fifth digits of the hands. Active fist closure was incomplete (approximately 80% of the normal range). Swelling, deformity, and contractures of the interphalangeal joints of the toes were also present, together with localized skin thickening over the first toe. The remaining joints were unremarkable, with preservation of the full range of motion.

**Patient 11**

*SCNN1B*(NM_000336.3):c.978C>A (p.Tyr326Ter), homozygous.

Clinical diagnosis: Pseudohypoaldosteronism, type I. Congenital ichthyosis? Hyperopia.

Status localis: Numerous pink miliary papules and excoriations were observed. Some lesions were covered with serous crusts and fine lamellar scales. The facial skin exhibited diffuse erythema with overlying desquamation. Thinning of the scalp hair, eyebrows, and eyelashes was noted. The nail plates were thin and brittle. The patient reported intense pruritus, particularly during the nighttime. The uninvolved skin was diffusely xerotic. The palms and soles were unaffected.

Skin biopsy: Histopathological examination demonstrated epidermal hyperkeratosis and parakeratosis with focal fibrin deposition admixed with neutrophils. Mild spongiosis was present. A superficial inflammatory infiltrate composed predominantly of lymphocytes with scattered neutrophils and occasional eosinophils was identified at the dermoepidermal junction. Immunohistochemical staining using antibodies against CD1a, Langerin, S100, and CD117 showed no positive staining within the inflammatory infiltrate. Periodic acid Schiff (PAS) and Grocott methenamine silver (GMS) stains revealed no evidence of fungal infection. The histopathological findings were consistent with seborrheic dermatitis. No histological evidence of Langerhans cell histiocytosis was identified in the examined specimen.

**Patient 12**

*FLG*(NM_002016.2):c.4420C>T (p.Arg1474Ter), heterozygous,

*FLG*(NM_002016.2):c.10558C>T (p.Gln3520Ter), heterozygous.

Clinical diagnosis: Ichthyosis vulgaris. Atopic dermatitis.

Status localis: Multiple pink miliary papules coalescing into moderately infiltrated papular plaques were present on the antecubital fossae and wrists. The uninvolved skin exhibited marked xerosis with fine lamellar desquamation and tightly adherent gray scales. Numerous follicular papules were also observed. Skin appendages were unremarkable.

**Patient 13**

*SPINK5*(NM_006846.4):c.1048C>T (p.Arg350Ter), homozygous.

Clinical diagnosis: Netherton syndrome. Astigmatism. Bilateral cryptorchidism.

Status localis: Diffuse erythema involved the entire cutaneous surface. The scalp was covered with densely adherent white and yellow scales. Fine lamellar desquamation was observed over the trunk and extremities on a background of generalized erythroderma. Lichenification was noted.

**Patient 14**

No genetic variants identified.

Clinical diagnosis: Palmoplantar keratoderma. Onychodystrophy. Autoimmune thyroiditis.

Status localis: The palmar surfaces demonstrated accentuated dermatoglyphics with thinning of the skin, focal punctate hyperkeratosis, and mild scaling. The plantar surfaces exhibited multifocal yellow hyperkeratosis; palpation revealed localized areas of induration. The plantar skin was thinned, with multiple foci of fine lamellar scaling. The nail plates showed marked onycholysis.

**Patient 15**

*LEMD3*(NM_014319.5):c.1963C>T (p.Arg655Ter), heterozygous.

Clinical diagnosis: Buschke-Ollendorff syndrome. Umbilical hernia. Myopia. Infantile idiopathic scoliosis.

Status localis: Multiple subcutaneous hemispherical papules and nodules with smooth surfaces and firm elastic consistency were present over the lumbar region and thighs. The lesions tended to coalesce. The overlying skin was of normal color with a faint yellowish hue visible through the surface. The lesions were non-tender on palpation. A normotrophic postoperative scar was present on the right buttock. The remaining skin was unremarkable apart from mild xerosis. Skin appendages were unaffected.

Skin biopsy: The epidermis was unremarkable. The dermis was mildly thinned. Skin appendages within the biopsy specimen included intact eccrine sweat glands and mildly atrophic hair follicles. Masson’s trichome and Van Gieson staining demonstrated a normal distribution of collagen and elastic fibers throughout the dermis. In the deep portion of the specimen, within the subcutaneous adipose tissue, extensive areas of fibrosis and hyalinization were identified. Inflammatory infiltrates were minimal. No cells with atypical morphological features were observed.

**Patient 16**

*NIPAL4*(NM_001099287.2):c.341C>A (p.Ala114Asp), homozygous.

Clinical diagnosis: Ichthyosis vulgaris.

Status localis: Fine- and medium-sized lamellar scaling was distributed over the entire cutaneous surface, with greater severity involving the distal portions of the extremities. The palms and soles demonstrated mild hyperkeratosis and hyperlinearity accompanied by linear fissures. The scalp was covered with densely adherent yellow crusts. The hair was dry but exhibited rapid growth.

Clinical photographs: Not available.

**Patient 17**

No genetic variants identified.

Clinical diagnosis: Verrucous nevus of the axillary region. Acanthosis nigricans.

Status localis: Hyperpigmented lesions ranging from brown to black in color were present on the face (perioral, periorbital, and periauricular regions), trunk, upper and lower extremities, and within all major flexural areas. The lesions exhibited a characteristic velvety surface with numerous papillomatous projections. Marked papillomatosis was particularly prominent in the axillary regions. The uninvolved skin was unremarkable, with normal elasticity and turgor. Skin appendages were unaffected.

Skin biopsy: Histopathological examination demonstrated papillomatous tissue covered by keratinizing stratified squamous epithelium with pronounced hyperkeratosis, papillomatosis, and hypergranulosis. The epithelial architecture was preserved, and no mitotic activity was identified. Focal acanthosis with marked hyperpigmentation of the basal keratinocyte layer was observed at the lesion periphery. The dermis showed vascular congestion and mild lymphocytic inflammatory infiltration. Correlation with the clinical findings indicated that the histopathological features were consistent with squamous papilloma arising in the setting of acanthosis nigricans.

**Patient 18**

*FLG*(NM_002016.2):c.3059C>G (p.Ser1020Ter), heterozygous.

Clinical diagnosis: Generalized pustular psoriasis.

Status localis: Multiple lenticular papules coalescing into pink plaques of varying size and configuration were present on the face and extremities, predominantly affecting the acral regions and extensor surfaces, on a background of marked xerosis. Individual lesions were covered with silvery scales. The psoriatic diagnostic triad (Brocq’s triad) was negative. Nail examination revealed a positive oil drop (“salmon patch”) sign and areas of onycholysis. Hair morphology was unremarkable, with preserved rapid growth. The Psoriasis Area and Severity Index (PASI) score was 15.7.

Clinical photographs: Not available.
