# Supplementary material for: Diagnostic utility of whole exome sequencing in children with suspected genodermatoses: a single-center retrospective study
Source: Front Med (Lausanne). 2026 Oct 7;13:1932248. doi: 10.3389/fmed.2026.1932248 (PMC13682972; doi:10.3389/fmed.2026.1932248)
Supplement: Supplementary file 2 [file Supplementaryfile2.docx]

Supplementary Material 2

Variants identified in the study and the ACMG criteria applied to their classification.

| **Patient** | **Genetic diagnosis, OMIM** | **Variant (hg 38)** | **Classification of variant, criteria and explanation** | **Zygosity** | **Reported** | **Allele frequency in gnomAD v4.1.1 (exomes+genomes)** |
| --- | --- | --- | --- | --- | --- | --- |
| Patient 1 | Noonan syndrome, 4 (610733) | *SOS1*(NM_005633.4):c.1654A>G (p.Arg552Gly) | Pathogenic variant  (PS4_mod, PM1_strong, PP3, PP2, PM6, PM2_supporting) by ClinGen Evidence Repository | het | Yes  (1) | 0.000001859 |
| Patient 2 | Epidermolysis bullosa simplex 5A, Ogna type (131950) | *PLEC*(NM_201378.4):c.12544G>A (p.Asp4182Asn) | Variant of Uncertain Significance (PM2_supporting – rare in control health population) | het | No | 0.00001550 |
| Patient 3 | Ichthyosis vulgaris (146700) | *FLG*(NM_002016.2):c.7339C>T (p.Arg2447Ter) | Likely pathogenic variant  (PVS1_strong – predicted to have frameshift without non-mediated decay, PM2_supporting – rare in control health population, PS4_mod) | het | Yes (2) | 0.003890 |
|  | Naxos disease (601214) | *JUP*(NM_002230.4):c.468G>A (p.Pro156=) | Pathogenic variant  (PM2_supporting – rare in control health population, PP3_strong – SpliceAI =0.87, PM3 – 2 affected patients with variant in homozygous state) | het | Yes (3), (4) | 0.000006209 |
|  |  | *JUP*(NM_002230.4):c.187C>T (p.Gln63Ter) | Likely pathogenic variant  (PVS1 – stop-gained (exon 2 of 14) at amino acid 63 of 745, predicted to trigger nonsense-mediated mRNA decay, PM2_supporting – rare in control health population) | het | No | - |
| Patient 4 | X-linked Olmsted syndrome (300918) | *MBTPS2*(NM_015884.4):c.656G>C (p.Arg219Thr) | Variant of Uncertain Significance (PM2_supporting - rare in control health population, PP3 – REVEL =0.91, AlphaMissense =1.0) | hemi | No | - |
| Patient 5 | Autosomal dominant epidermolytic hyperkeratosis 2A (620150) | *KRT10*(NM_000421.5):c.467G>A (p.Arg156His) | Pathogenic variant  (PS3 – variant cause aberrant keratin filament networks (5), PM2_supporting – rare in control health population, PM5 – c.466C>T, p.Arg156Cys described as Pathogenic (6), PS4_mod) | het | Yes (7) | - |
| Patient 7 | Epidermolytic hyperkeratosis, 1 (113800) | *KRT1*(NM_006121.4):c.583A>T (p.Ile195Phe) | Variant of Uncertain Significance  (PM2_supporting – rare in control health population, PP3_strong – REVEL =0.91, AlphaMissense =0.99) | het | No | - |
| Patient 8 | Netherton syndrome (256500) | *SPINK5*(NM_006846.4):c.1048C>T (p.Arg350Ter) | Pathogenic variant  (PVS1 – stop-gained (exon 12 of 33) at amino 350 of 1064, predicted to trigger nonsense-mediated mRNA decay, PM2_supporting – rare in control health population, PM3_strong – (8), (9)) | hom | Yes  (10) | 0.00004776 |
| Patient 9 | X-linked ichthyosis (308100) | Deletion with genomic coordinates chrX:7050166-7804358 | Pathogenic copy number variant  (1A – Contains protein-coding elements, 2A – Complete overlap of an established HI gene/genomic region, 4L – Statistically significant increase amongst observations in cases (with a consistent, specific, well-defined phenotype) compared to controls) by ClinGen CNV Pathogenicity Calculator | hemi | Yes (11) | - |
| Patient 10 | Autosomal recessive congenital ichthyosis, 6 (612281) | *NIPAL4*(NM_001099287.2):c.586G>A (p.Gly196Arg) | Variant of Uncertain Significance  (PM2_supporting – rare in control health population, PP3 – REVEL =0.86, SpliceAI =0.97) | hom | No | - |
| Patient 11 | Pseudohypoaldosteronism, type IB2, autosomal recessive (620125) | *SCNN1B*(NM_000336.3):c.978C>A (p.Tyr326Ter) | Likely pathogenic variant  (PVS1 – stop-gained (exon 6 of 13) at amino acid 326 of 640, predicted to trigger nonsense-mediated mRNA decay, PM2_supporting – rare in control health population) | hom | No | 0.000001859 |
| Patient 12 | Ichthyosis vulgaris (146700) | *FLG*(NM_002016.2):c.4420C>T (p.Arg1474Ter) | Likely pathogenic variant  (PVS1_strong – stop-gained (exon 3 of 3) at amino acid 1474 of 4061, NMD not predicted, PM2_supporting – rare in control health population, PS4_mod) | het | Yes (2) | 0.0003533 |
|  |  | *FLG*(NM_002016.2):c.10558C>T (p.Gln3520Ter) | Likely pathogenic variant  (PVS1_strong – stop-gained (exon 3 of 3) at amino acid 1474 of 4061, NMD not predicted, PM2_supporting – rare in control health population, PS4_mod) | het | Yes (12) | 0.00002048 |
| Patient 13 | Netherton syndrome (256500) | *SPINK5*(NM_006846.4):c.1048C>T (p.Arg350Ter) | Pathogenic variant  (PVS1 – stop-gained (exon 12 of 33) at amino 350 of 1064, predicted to trigger nonsense-mediated mRNA decay, PM2_supporting – rare in control health population, PM3_strong - (8), (9)) | hom | Yes  (10) | 0.00004776 |
| Patient 15 | Buschke-Ollendorff syndrome (166700) | *LEMD3*(NM_014319.5):c.1963C>T (p.Arg655Ter) | Pathogenic variant  (PVS1 – Stop-gained (exon 7 of 13) at amino acid 655 of 911, predicted to trigger nonsense-mediated mRNA decay, PM2_supporting – rare in control health population) | het | Yes (13) | 0.000002482 |
| Patient 16 | Autosomal recessive congenital ichthyosis, 6 (612281) | *NIPAL4*(NM_001099287.2):c.341C>A (p.Ala114Asp) | Likely pathogenic variant  (PM2_supporting – rare in control health population, PP3 – REVEL =0.77, AlphaMissense =0.93, PM3_strong – (14) | hom | Yes (15) | 0.001112 |
| Patient 18 | Ichthyosis vulgaris (146700) | *FLG*(NM_002016.2):c.3059C>G (p.Ser1020Ter) | Likely pathogenic variant  (PVS1_strong – stop-gained (exon 3 of 3) at amino acid 1020 of 4061, NMD not predicted, PM2_supporting – rare in control health population, PS4_mod) | het | Yes (16) | 0.0000855 |

**References**

1.	Zenker M, Horn D, Wieczorek D, Allanson J, Pauli S, van der Burgt I, Doerr H, Gaspar H, Hofbeck M, Gillessen‐Kaesbach G, et al. SOS1 is the second most common Noonan gene but plays no major role in cardio‐facio‐cutaneous syndrome. *J Med Genet* (2007) 44:651–656. doi: 10.1136/jmg.2007.051276

2.	Sandilands A, Terron-Kwiatkowski A, Hull PR, O’Regan GM, Clayton TH, Watson RM, Carrick T, Evans AT, Liao H, Zhao Y, et al. Comprehensive analysis of the gene encoding filaggrin uncovers prevalent and rare mutations in ichthyosis vulgaris and atopic eczema. *Nat Genet* (2007) 39:650–654. doi: 10.1038/ng2020

3.	Oktem A, Doolan BJ, Akay BN, Onoufriadis A, Okcu Heper A, Kocak O, Ersoy-Evans S, McGrath JA. Autosomal recessive mutations in plakoglobin and risk of cardiac abnormalities. *Clin Exp Dermatol* (2020) 45:654–657. doi: 10.1111/ced.14201

4.	Cabral RM, Liu L, Hogan C, Dopping-Hepenstal PJC, Winik BC, Asial RA, Dobson R, Mein CA, Baselaga PA, Mellerio JE, et al. Homozygous Mutations in the 5′ Region of the JUP Gene Result in Cutaneous Disease but Normal Heart Development in Children. *J Invest Dermatol* (2010) 130:1543–1550. doi: 10.1038/jid.2010.7

5.	Cheng J, Syder AJ, Yu QC, Letai A, Paller AS, Fuchs E. The genetic basis of epidermolytic hyperkeratosis: a disorder of differentiation-specific epidermal keratin genes. *Cell* (1992) 70:811–819. doi: 10.1016/0092-8674(92)90314-3

6.	Cheraghlou S, Atzmony L, Roy SF, McNiff JM, Choate KA. Mutations in KRT10 in epidermolytic acanthoma. *J Cutan Pathol* (2020) 47:524–529. doi: 10.1111/cup.13664

7.	Frommherz L, Giehl K, Hofmann J, Huebner S, Kiekbusch K, Sabkova T, Süßmuth K, Alter S, Tantcheva‐Poór I, Ott H, et al. Epidermolytic ichthyosis: Clinical spectrum and burden of disease in a large German cohort. *J Eur Acad Dermatol Venereol* (2025) 39:1028–1037. doi: 10.1111/jdv.20096

8.	Hannula-Jouppi K, Laasanen S-L, Ilander M, Furio L, Tuomiranta M, Marttila R, Jeskanen L, Häyry V, Kanerva M, Kivirikko S, et al. Intrafamily and Interfamilial Phenotype Variation and Immature Immunity in Patients With Netherton Syndrome and Finnish SPINK5 Founder Mutation. *JAMA Dermatol* (2016) 152:435–442. doi: 10.1001/jamadermatol.2015.5827

9.	Nartisa I, Kirsteina R, Neiburga KD, Zigure S, Ozola L, Grantina I, Micule I, Murmane D, Slisere B, Gailite L, et al. Clinical and genetic characterization of Netherton syndrome due to SPINK5 founder variant in Latvian population. *Pediatr Allergy Immunol* (2023) 34:e13937. doi: 10.1111/pai.13937

10.	Bitoun E, Chavanas S, Irvine AD, Lonie L, Bodemer C, Paradisi M, Hamel-Teillac D, Ansai S, Mitsuhashi Y, Taïeb A, et al. Netherton Syndrome: Disease Expression and Spectrum of SPINK5 Mutations in 21 Families. *J Invest Dermatol* (2002) 118:352–361. doi: 10.1046/j.1523-1747.2002.01603.x

11.	Diociaiuti A, Angioni A, Pisaneschi E, Alesi V, Zambruno G, Novelli A, El Hachem M. X-linked ichthyosis: Clinical and molecular findings in 35 Italian patients. *Exp Dermatol*. (2019) 28(10):1156-1163. doi:10.1111/exd.13667

12.	Paul AA, Szulc NA, Kobiela A, Brown SJ, Pokrzywa W, Gutowska-Owsiak D. In silico analysis of the profilaggrin sequence indicates alterations in the stability, degradation route, and intracellular protein fate in filaggrin null mutation carriers. *Front Mol Biosci* (2023) 10:1105678. doi: 10.3389/fmolb.2023.1105678

13.	Zhang Y, Castori M, Ferranti G, Paradisi M, Wordsworth BP. Novel and recurrent germline LEMD3 mutations causing Buschke-Ollendorff syndrome and osteopoikilosis but not isolated melorheostosis. *Clin Genet* (2009) 75:556–561. doi: 10.1111/j.1399-0004.2009.01177.x

14.	Lefèvre C, Bouadjar B, Karaduman A, Jobard F, Saker S, Ozguc M, Lathrop M, Prud’homme J-F, Fischer J. Mutations in ichthyin a new gene on chromosome 5q33 in a new form of autosomal recessive congenital ichthyosis. *Hum Mol Genet* (2004) 13:2473–2482. doi: 10.1093/hmg/ddh263

15.	Steiner CE, Puzzi MB, Marques-de-Faria AP, de Oliveira Sobrinho RP, Gil-da-Silva-Lopes VL, Moreno CA. A Series of Patients with Genodermatoses in a Reference Service for Rare Diseases: Results from the Brazilian Rare Genomes Project. *Genes* (2025) 16:522. doi: 10.3390/genes16050522

16.	Salava A, Salo V, Remitz A. Keratosis pilaris and filaggrin loss‐of‐function mutations in patients with atopic dermatitis – Results of a Finnish cross‐sectional study. *J Dermatol* (2022) 49:928–932. doi: 10.1111/1346-8138.16477
